# Relationship Between Localization Acuity and Spatial Release from Masking

**DOI:** 10.3390/audiolres16040108

**Published:** 2026-07-28

**Authors:** Nirmal Srinivasan

**Affiliations:** Department of Speech-Language Pathology and Audiology, Towson University, Towson, MD 21252, USA; nsrinivasan@towson.edu

**Keywords:** free-field localization, spatial release from masking, spatial cue availability

## Abstract

**Background/Objectives:** Spatial hearing enables listeners to segregate competing sound sources in complex acoustic environments. Two key components of spatial hearing are localization acuity and spatial release from masking (SRM). Although both rely on similar binaural cue processing, their relationship remains unclear. The present study aimed to investigate localization acuity using frequency-specific and broadband stimuli and to determine how these measures relate to SRM in young normal-hearing listeners. **Methods:** Forty-five normal-hearing participants completed free-field localization and speech identification tasks using a multi-loudspeaker array. Localization acuity was assessed using one-third octave low-frequency (500 Hz), high-frequency (5000 Hz), and broadband noise stimuli. SRM was measured using a Coordinate Response Measure (CRM) speech task under colocated and spatially separated conditions (±30°). Localization performance was quantified using percent correct and root-mean-square (RMS) error, and relationships between localization acuity and SRM were examined using correlation analyses. **Results:** Localization performance varied significantly with stimulus bandwidth, with broadband stimuli yielding the highest accuracy, followed by low- and high-frequency conditions. Listeners demonstrated robust SRM (~5.6 dB). Moderate negative correlations were observed between RMS error and SRM across all stimulus conditions. Although the broadband condition yielded numerically larger correlation coefficients, differences among correlations were not statistically significant. **Conclusions:** Localization acuity was moderately associated with SRM across stimulus conditions. Although numerically larger correlations were observed for broadband stimuli, these differences were not statistically significant. The findings suggest that localization acuity represents one of several factors contributing to spatial hearing performance in complex listening environments.

## 1. Introduction

Humans routinely navigate complex acoustic environments in which multiple sound sources compete for perceptual representation. A key factor enabling successful listening in such environments is the ability to exploit spatial cues to segregate and attend to target sounds. Two constructs are central to this capability: localization acuity, which reflects the precision with which listeners can detect or discriminate sound source locations, and spatial release from masking (SRM), which refers to the improvement in speech intelligibility or signal detection when target and masker sources are spatially separated rather than co-located. Although both phenomena depend on spatial hearing mechanisms, the extent to which they are directly related remains an open question. The present manuscript investigates the relationship between localization acuity and SRM, with the goal of clarifying whether individual differences in spatial resolution predict the ability to benefit from spatial separation in multitalker or noisy environments.

Localization acuity is commonly quantified using measures such as the minimum audible angle (MAA) or localization error in free-field listening tasks. These metrics capture the auditory system’s sensitivity to binaural cues, including interaural time differences (ITDs) and interaural level differences (ILDs), as well as monaural spectral cues shaped by the outer ear [1,2]. High localization acuity reflects precise encoding and integration of these cues across frequency channels and spatial positions. Importantly, localization performance varies across listeners due to factors such as age-related changes, hearing impairment, and experience, suggesting that spatial processing is subject to individual variability [3,4].

Free-field localization studies provide critical ecological insight into how listeners use spatial cues under realistic conditions, complementing more constrained laboratory measures such as MAA. Horizontal-plane sound localization studies using multi-loudspeaker arrays have demonstrated that localization performance depends not only on cue sensitivity but also on stimulus bandwidth and spatial configuration [2,5]. Their findings highlight systematic patterns of localization bias and variability across azimuth, reflecting the complex integration of binaural and spectral information in natural listening environments [1,2]. Similarly, ref. [5] investigated sound localization abilities in both normal-hearing listeners and individuals with hearing impairment in a free-field setup. Their work demonstrated that accurate localization depends strongly on access to symmetric binaural cues, and that disruptions to these cues can significantly degrade spatial accuracy. Together, these studies emphasize that localization acuity measured in free-field contexts captures a rich and multidimensional aspect of spatial hearing that may be particularly relevant for understanding real-world listening performance.

Spatial release from masking, in contrast, is typically assessed in paradigms involving speech recognition in the presence of competing maskers. When the masker is spatially separated from the target, listeners often exhibit substantial improvements—sometimes exceeding 10 dB in signal-to-noise ratio—compared to co-located conditions [6,7]. This benefit arises from both energetic and informational processes. Energetic unmasking occurs through peripheral separation of sound energy at the ears, while informational masking reductions involve higher-level perceptual and cognitive processes that facilitate stream segregation and selective attention [8,9]. The contribution of spatial cues to SRM is widely documented, yet the mechanisms underlying inter-individual variability are less well understood.

Given that both localization acuity and SRM rely on accurate spatial cue processing, a close relationship between the two might be expected [10,11]. Indeed, some theoretical frameworks propose that fine spatial discrimination abilities should support more effective segregation of competing sound sources, thereby enhancing SRM [11]. For example, listeners with superior sensitivity to ITDs and ILDs may be better equipped to exploit spatial differences between target and masker, improving both perceptual separation and attentional focus. Furthermore, neural coding of spatial cues in the auditory brainstem and cortex provides a shared substrate for both localization and spatial unmasking, suggesting overlapping physiological mechanisms [10].

However, empirical evidence for a strong link between localization acuity and SRM has been mixed. Some studies report modest correlations between localization performance and speech-in-noise benefits, particularly in populations with hearing impairment or altered binaural processing [12,13]. Other investigations, however, find weak or inconsistent relationships, indicating that good localization ability does not necessarily guarantee large spatial masking release [14]. These discrepancies may reflect the multifaceted nature of SRM, which involves not only spatial resolution but also cognitive factors such as attention, working memory, and linguistic processing [15].

One explanation for the limited correspondence between localization acuity and SRM is that the tasks used to measure these constructs tap into different aspects of spatial hearing. Localization tasks typically require explicit judgments of sound direction, often using brief, broadband signals presented in quiet or controlled free-field environments [5,16]. In contrast, SRM tasks involve continuous or temporally complex stimuli presented in challenging acoustic environments, requiring listeners to segregate and track a target stream over time [17,18]. Thus, localization acuity may capture static spatial resolution, whereas SRM reflects the use of spatial information in dynamic, attentionally demanding contexts. This distinction suggests that shared sensory encoding may be necessary but not sufficient for SRM.

Another important consideration is that SRM includes both binaural unmasking and spatial attention mechanisms. Binaural unmasking arises from the comparison of signals across the two ears and can be quantified through measures such as the binaural masking level difference [19]. Spatial attention, on the other hand, involves the selective enhancement of target signals at higher cortical levels and may depend on cognitive control and experience [11]. Localization acuity, even when assessed in realistic free-field conditions [5], may not fully capture these attentional and cognitive processes, thereby limiting its predictive power for SRM outcomes.

The relationship between localization acuity and SRM also has important implications for clinical populations, including individuals with hearing loss and users of hearing aids or cochlear implants. For example, hearing-impaired listeners often exhibit reduced localization accuracy due to degraded binaural cues, which may also limit their ability to benefit from spatial separation in noisy environments [20]. However, assistive devices can differentially affect localization and SRM, sometimes improving one while leaving the other unchanged [21]. Similarly, cochlear implant and bimodal hearing studies have demonstrated improvements in localization accuracy without proportional gains in speech-in-noise performance, or vice versa, indicating that these outcomes depend on partially distinct mechanisms [22]. Understanding the extent to which these abilities are linked could inform the design of interventions aimed at enhancing spatial hearing, such as spatial cue preservation strategies or auditory training programs.

In addition, recent advances in auditory neuroscience and computational modeling have highlighted the complexity of spatial hearing processes. Models of auditory scene analysis emphasize the integration of spatial, spectral, and temporal cues in forming robust perceptual representations [23]. These models suggest that SRM depends on a combination of low-level sensory encoding and higher-level grouping principles, which may not be fully captured by traditional measures of localization acuity. Consequently, examining the relationship between these constructs provides an opportunity to test and refine theoretical accounts of spatial hearing.

In summary, while localization acuity and spatial release from masking both rely on spatial hearing mechanisms, their relationship is not yet fully understood. Clarifying this relationship is critical for advancing theoretical models of auditory perception and for developing effective clinical interventions to improve communication in complex listening environments. The present study is motivated by the need to better understand how the fidelity of spatial cue processing across different frequency regions contributes to functional listening benefits in complex acoustic environments. Although localization acuity has traditionally been measured using broadband stimuli, real-world listening often involves signals with restricted spectral content, and the auditory system relies differentially on low- and high-frequency cues. Low-frequency sounds primarily convey interaural time differences (ITDs), which are critical for azimuthal localization, whereas high-frequency sounds provide interaural level differences (ILDs) and spectral cues shaped by the pinna [1,2]. However, the extent to which localization acuity derived from frequency-restricted stimuli reflects spatial processing abilities that support spatial release from masking (SRM) remains unclear. By systematically measuring localization acuity using one-third octave low-frequency-centered noise, one-third octave high-frequency-centered noise, and broadband noise in young normal-hearing listeners, the current study aims to disentangle the contributions of distinct binaural cue regimes to spatial resolution. Relating these measures to SRM will provide insight into whether sensitivity to specific spatial cues—or the integration of cues across frequency—better predicts an individual’s ability to benefit from spatial separation in multitalker environments. This approach offers a more nuanced characterization of spatial hearing by linking controlled psychophysical measures of localization acuity with ecologically relevant outcomes of speech perception in noise.

## 2. Methods

### 2.1. Participants

Forty-five female listeners (ages 20–25 years) with normal hearing participated in the study. All listeners had pure-tone air-conduction thresholds ≤ 20 dB HL at octave frequencies from 250 to 8000 Hz in both ears. None reported a history of otologic or neurological disorders. Participants provided informed consent prior to testing and were compensated for their time. All procedures were approved by Towson University’s institutional review board.

### 2.2. Apparatus and Test Environment

Speech identification and localization experiments were conducted in a double-walled sound-attenuating booth at the Spatial Hearing and Auditory Perception (SHAPE) laboratory at Towson University. Stimuli were presented in free field using Orb Audio Mod1X spherical loudspeakers arranged along the horizontal plane positioned at ear level. The array consisted of 13 loudspeakers spaced at 15° intervals, spanning −90° to +90° azimuth relative to the listener’s midline. Participants were instructed to orient their head toward the 0° loudspeaker before each trial and were reminded periodically by the experimenter. No head stabilization device (e.g., chin rest) was used. Stimulus generation and presentation were controlled using MATLAB (R2020a, MathWorks, Natick, MA, USA) and multichannel audio interfaces (Ashly NE8250 power amplifiers equipped with OPDante option). All stimuli were calibrated using a sound level meter to ensure consistent output levels across loudspeakers.

### 2.3. Stimuli

#### 2.3.1. Speech Identification Task

Three male talkers from the Coordinate Response Measure (CRM; [24]) were used for the target and masker sentences. All sentences in the CRM corpus have the form “Ready [CALL SIGN] go to [COLOR] [NUMBER] now”. There are eight possible call signs (Arrow, Baron, Charlie, Eagle, Hopper, Laker, Ringo, and Tiger), four colors (Blue, Red, White, and Green), and eight numbers (1–8). All possible combinations of the call signs, colors, and numbers were used. On each trial, the listener was presented with a CRM sentence in the presence of two similar CRM sentences. The goal was to attend the sentence identified by the call sign “Charlie”. The maskers were chosen to have unique call signs, and the target and the masker talkers, color, and number varied from trial to trial.

#### 2.3.2. Localization Task

Three classes of noise stimuli were used to assess localization acuity:

1. Low-frequency narrowband noise: One-third octave noise centered at 500 Hz
2. High-frequency narrowband noise: One-third octave noise centered at 5000 Hz
3. Broadband noise: Gaussian noise spanning 200–6000 Hz

The low-frequency and high-frequency narrowband noises were chosen for reasons outlined in [5]. In short, frequencies less than 1000 Hz do not generate a large ILD and hence, ITD is primarily used to localize sounds in that frequency range. Also, for frequencies greater than 1500 Hz, the ITD information is ambiguous and hence, ILD is primarily used to localize sounds [25]. The broadband signal was used so that both ITD and ILD cues were present in the signal.

### 2.4. Procedure

#### 2.4.1. Speech Identification Task

The experimental session always started with a quiet threshold estimation, which was defined as the level required to obtain the 50% correct point on the psychometric function [26]. Quiet thresholds were obtained using a one-up, one-down adaptive procedure, based on whether or not the color and number of the target sentence were reported correctly. The speech level was initially set 20 dB above the PTA (pure-tone average of audiometric thresholds for 0.5, 1, 2, and 4 kHz). The level of the target sentence was reduced by 5 dB after every correct trial and increased by 5 dB after every incorrect trial until three reversals of direction occurred, at which point step size was reduced to 1 dB from the fourth reversal onwards. Each track had 10 reversals, and the threshold was estimated based on the average of the last six reversals. Three estimates of threshold in quiet were obtained and the average of the last two threshold estimates was used to set the target level for the speech identification testing in the presence of the maskers.

Two spatial separations were used for the speech identification task. In the colocated condition, both the target and the masking speech were presented from the 0° loudspeaker. In the spatially separated condition, the target speech was presented from the 0° loudspeaker while the masking speech was presented from the +30° and −30° loudspeakers. The target speech level was fixed at 20 dB above the quiet threshold, and the masker level was varied adaptively to estimate the target-to-masker ratio (dB) required to obtain the 50% correct point on the psychometric function. The step sizes and the number of reversals were similar to the quiet threshold estimation explained earlier.

Responses in the speech identification task were collected using a closed-set response interface displayed on a touch screen computer monitor. Following each trial, participants selected the color–number combination for the call sign “Charlie”. Feedback (“Correct” or “Incorrect”) was provided after every response. Each participant completed two threshold tracks for each spatial condition.

#### 2.4.2. Localization Acuity

Localization acuity was measured using a single-interval identification task. On each trial, a stimulus was presented randomly from one of the thirteen loudspeaker locations. The level of the noise stimulus was fixed at 20 dB above the average of pure-tone audiometric thresholds across 0.25, 0.5, 1, and 2 kHz (20 dB SL). Participants were instructed to indicate the perceived location by selecting the corresponding loudspeaker number displayed on a touch screen computer monitor that was located about 2 ft below their head. Feedback (“Correct” or “Incorrect”) was provided after every response.

Each class of the noise stimuli (low-frequency, high-frequency, broadband) was tested in separate blocks. No sound was presented from the +90° and −90° loudspeakers to reduce any edge effects [27]. Each location was presented 10 times per condition, in two separate blocks, resulting in 220 trials per noise condition. The order of the conditions was randomized across participants.

### 2.5. Dependent Measures

Spatial release from masking (SRM) was calculated as the difference between the colocated threshold and the spatially separated threshold. Higher SRM values indicate greater benefit from spatially separating the target from the masker. For the localization task, mean localization accuracy was computed as the proportion of correctly identified loudspeaker locations relative to the number of presentations multiplied by 100. This measure provided an intuitive index of overall localization performance, reflecting the listener’s ability to accurately map perceived auditory space onto the physical loudspeaker array. For location-specific analyses, RMS error was calculated separately for each loudspeaker location using the responses obtained for that location. Specifically, the RMS error for a given azimuth was computed across the 20 presentations obtained at that location (10 presentations in each of two test blocks) using the following formula:

$$rms\;\left({}^{\circ}\right)=\sqrt{\frac{\sum_{i=1}^{20}{\left({Stimulus}_{i}-{Response}_{i}\right)}^{2}}{20}}\;$$

Mean RMS error at each azimuth was then averaged across listeners for subsequent statistical analyses. RMS error is sensitive to near-miss responses and therefore provides a more nuanced characterization of spatial accuracy across the full range of stimulus locations.

### 2.6. Data Analysis

Localization acuity metrics (RMS error and localization accuracy) were computed separately for each stimulus condition. Because the primary goal was to evaluate relationships between participant-level measures of localization acuity and SRM, analyses were conducted using participant-level summary statistics. Repeated-measures analyses of variance (ANOVA) were conducted to assess the effect of stimulus type (low-frequency, high-frequency, broadband) on localization performance. To examine the relationship between localization acuity and SRM, correlation analyses (Pearson’s *r*) were performed between SRM and RMS error at 30° for each of the three noise conditions. Differences between correlation coefficients were evaluated using Steiger’s Z tests [28]. Correlations involving the same sample of participants were statistically compared to determine whether localization measures obtained at ±30° azimuth were more strongly associated with SRM than localization measures that averaged across all loudspeaker locations. Statistical significance was evaluated at α = 0.05, with corrections applied for multiple comparisons where appropriate.

## 3. Results

**Figure 1:**
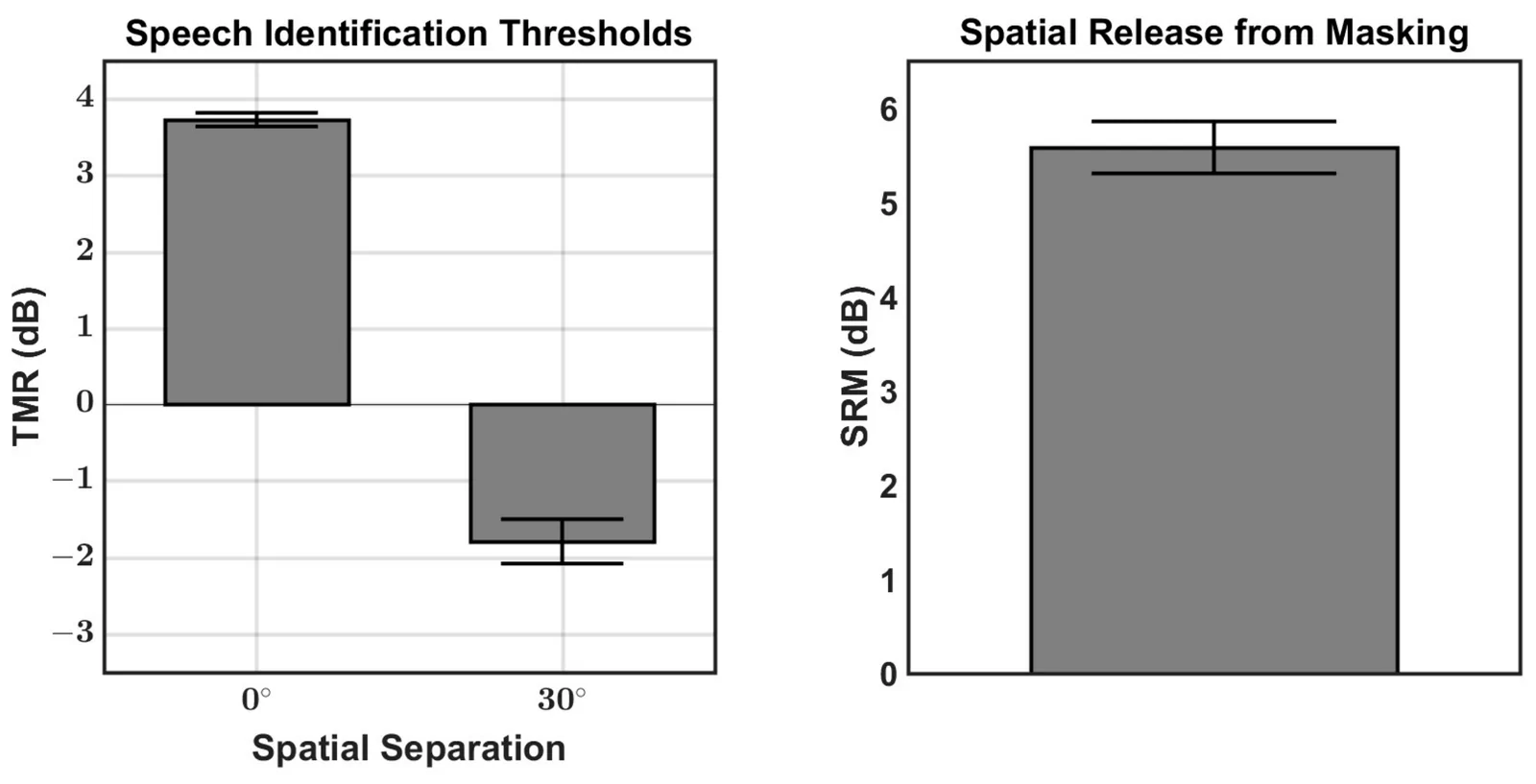
Left panel displays the speech identification threshold (the target-to-masker ratio (TMR) required to identify 50% of the key words) at the two spatial separations, while right panel displays spatial release from masking (SRM; difference between the colocated and the spatially separated speech identification thresholds). Error bars reflect ±1 standard error of the mean (SEM).

In Figure 1, panel a shows the average speech identification thresholds (±1 standard error of mean) at the two spatial separations tested, while panel b shows the average amount of spatial release from masking (±1 standard error of mean). A paired-samples *t*-test was conducted to investigate the effect of spatial separation on speech identification thresholds. As expected, there was a significant difference between speech identification thresholds at the two spatial separations tested (*t*(44) = 20.21, *p* < 0.001, Cohen’s d = 3.61, 95% CI [2.75, 4.46], indicating a large effect) with the spatially separated condition having a significantly lower (more favorable) speech identification threshold (*M* = −1.85 dB, *SD* = 1.93 dB) than the colocated condition (*M* = 3.72 dB, *SD* = 0.64 dB). By spatially separating the maskers from the target, on average, the listeners obtained 5.56 dB (*SD* = 1.85) release from masking.

**Figure 2:**
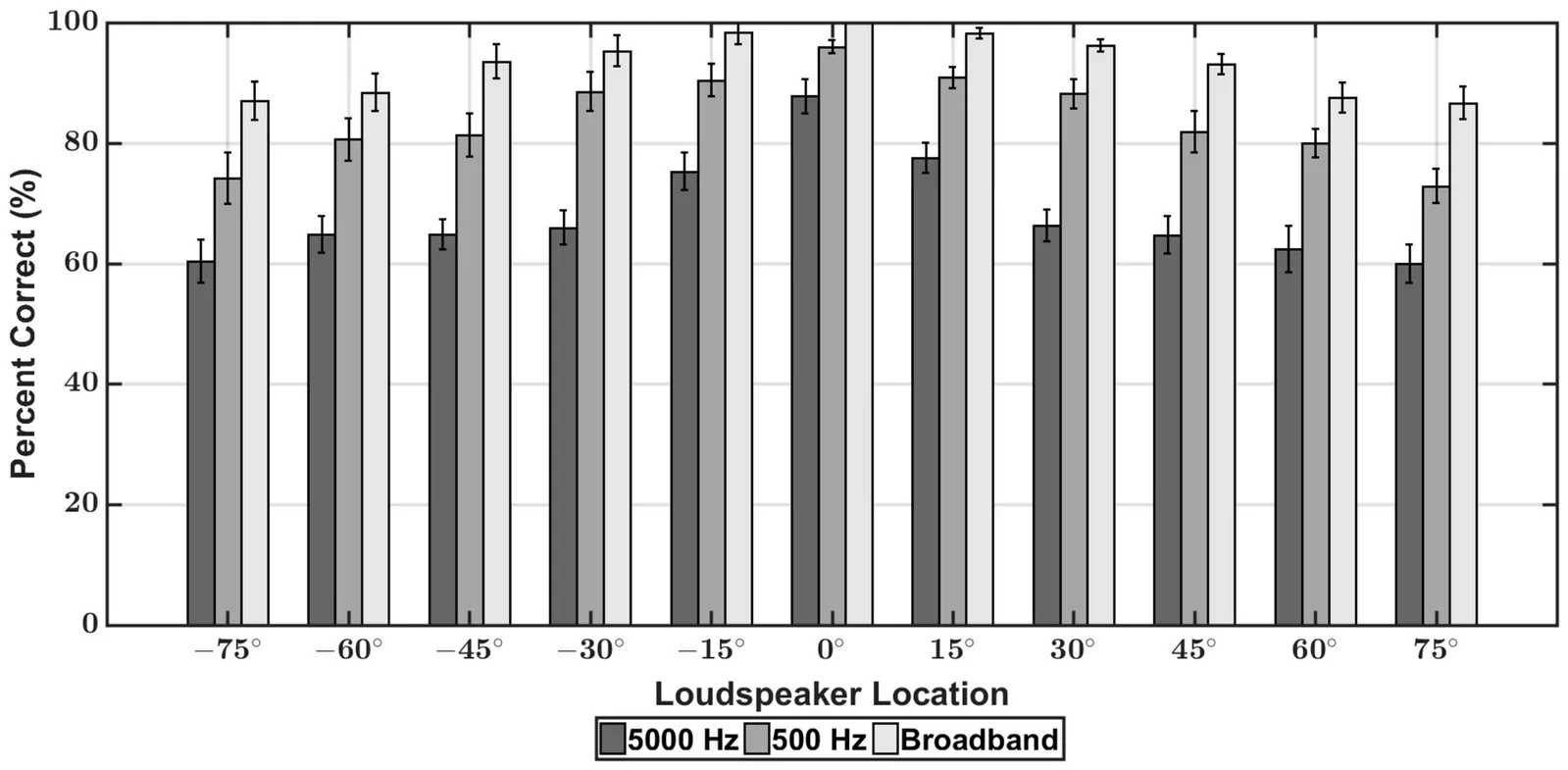
Mean localization accuracy (percent correct (%)) at various loudspeaker locations for the three types of noise stimuli used in this experiment. Error bars reflect ±1 standard error of the mean (SEM).

Figure 2 shows the mean localization accuracy (±1 standard error of mean) for the three types of noise stimuli presented at various spatial locations in this experiment. A repeated measures factorial ANOVA was conducted to investigate the effects of noise stimuli (broadband, low frequency, and high frequency) and spatial locations (0°, ±15°, ±30°, ±45°, ±60°, and ±75°) on mean localization accuracy. There was a significant main effect of noise stimuli on mean localization accuracy (*F*(2,88) = 108.91, *p* < 0.001, *partial η*^2^ = 0.712, indicating a large effect). Post hoc analyses with Bonferroni correction indicated that the mean localization accuracy was ordered as follows: broadband > low-frequency narrowband noise > high-frequency narrowband noise. Mauchly’s test indicated that the assumption of sphericity had been violated for spatial locations and therefore degrees of freedom were corrected using Greenhouse–Geisser estimates of sphericity (*χ*^2^(54) = 180.04, *p* < 0.001, *ε* = 0.52). There was a significant main effect of spatial location on mean localization accuracy (*F*(5.16, 227.12) = 55.03, *p* < 0.001, *partial η*^2^ = 0.556, indicating a large effect). Post hoc analyses with Bonferroni correction indicated that the mean localization accuracy was the highest when the signal was presented from the 0° spatial location and the mean localization accuracy systematically reduced as the presentation location moved away from the 0° spatial location. Mauchly’s test indicated that the assumption of sphericity had been violated for the interaction between noise stimuli and spatial locations and therefore degrees of freedom were corrected using Greenhouse–Geisser estimates of sphericity (*χ*^2^(209) = 361.19, *p* < 0.001, *ε* = 0.57). There was a significant interaction effect of noise stimuli and spatial locations on mean localization accuracy (*F*(11.31, 497.72) = 5.67, *p* < 0.001, *partial η*^2^ = 0.114, indicating a moderate effect). Simple effect analyses with Bonferroni correction indicated that the mean localization accuracy for all eleven spatial locations was ordered as follows: broadband > low-frequency narrowband noise > high-frequency narrowband noise.

**Figure 3:**
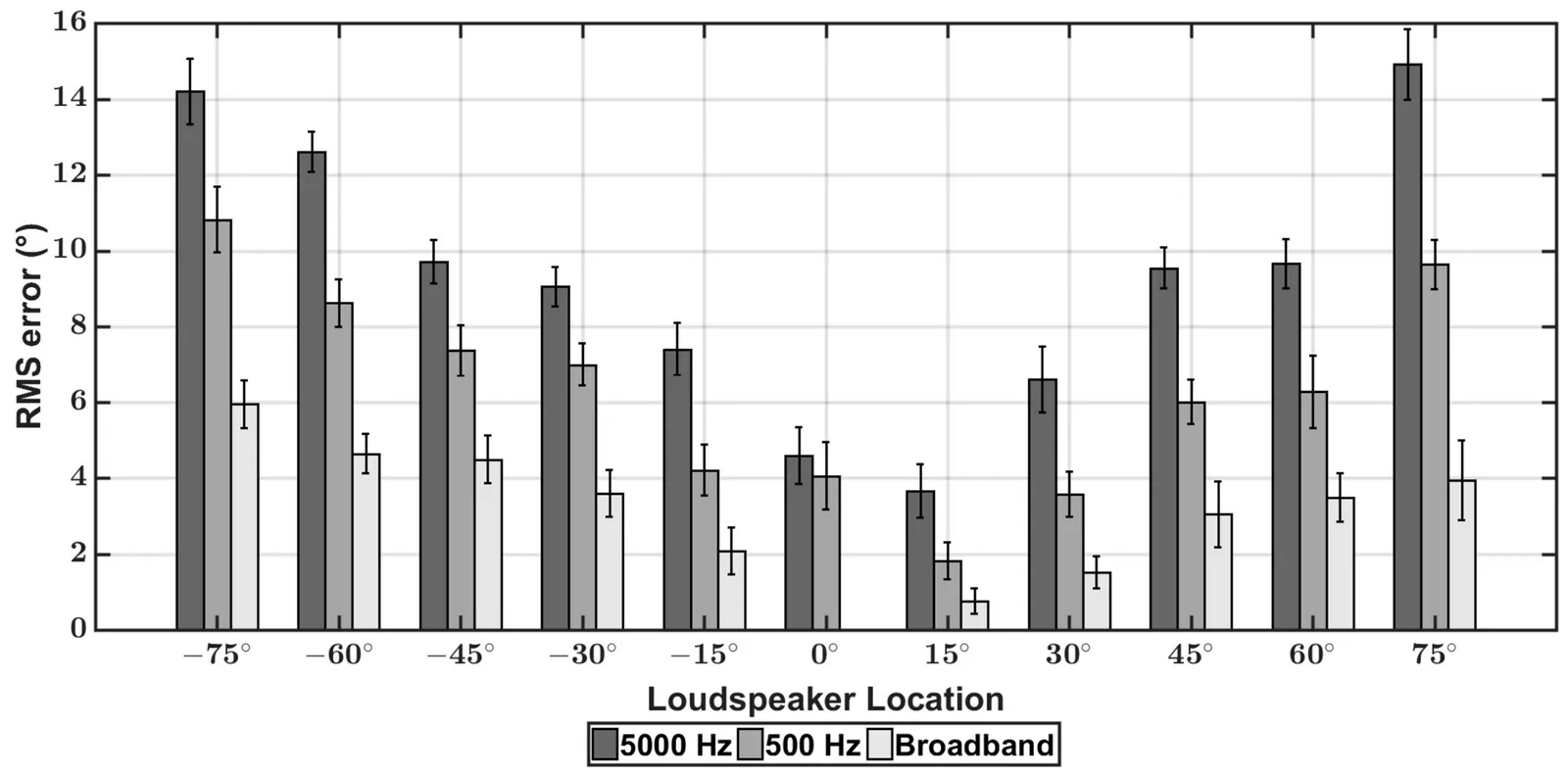
Mean RMS error (°) at various loudspeaker locations for the three types of noise stimuli used in this experiment. Error bars reflect ±1 standard error of the mean (SEM).

Figure 3 shows the mean RMS error (±1 standard error of mean) for the three types of noise stimuli presented at various spatial locations in this experiment. A repeated measures factorial ANOVA was conducted to investigate the effects of noise stimuli (broadband, low frequency, and high frequency) and spatial locations (0°, ±15°, ±30°, ±45°, ±60°, and ±75°) on RMS errors. There was a significant main effect of noise stimuli on mean RMS error (*F*(2,88) = 87.99, *p* < 0.001, *partial η^2^* = 0.667, indicating a large effect). Post hoc analyses with Bonferroni correction indicated that the RMS errors were ordered as follows: broadband < low-frequency narrowband noise < high-frequency narrowband noise. Mauchly’s test indicated that the assumption of sphericity had been violated for spatial locations and therefore degrees of freedom were corrected using Greenhouse–Geisser estimates of sphericity (*χ*^2^(54) = 199.79, *p* < 0.001, ε = 0.48). There was a significant main effect of spatial location on RMS errors (*F*(4.81, 211.49) = 47.75, *p* < 0.001, *partial η^2^* = 0.520, indicating a large effect). Post hoc analyses with Bonferroni correction indicated that the RMS error was the lowest when the signal was presented from the 0° spatial location and the RMS errors systematically increased as the presentation location moved away from the 0° spatial location. Mauchly’s test indicated that the assumption of sphericity had been violated for the interaction between noise stimuli and spatial locations and therefore degrees of freedom were corrected using Greenhouse–Geisser estimates of sphericity (*χ*^2^(209) = 410.35, *p* < 0.001, *ε* = 0.52). There was a significant interaction effect of noise stimuli and spatial locations on RMS errors (*F*(10.36, 454.76) = 6.12, *p* < 0.001, *partial η^2^* = 0.122, indicating a moderate effect). Simple effect analyses with Bonferroni correction indicated that the RMS errors for all spatial locations other than the 0° spatial location were ordered as follows: broadband < low-frequency narrowband noise < high-frequency narrowband noise. At the 0° spatial location, there was no significant difference in the RMS errors between the low-frequency and high-frequency noise stimuli.

**Figure 4:**
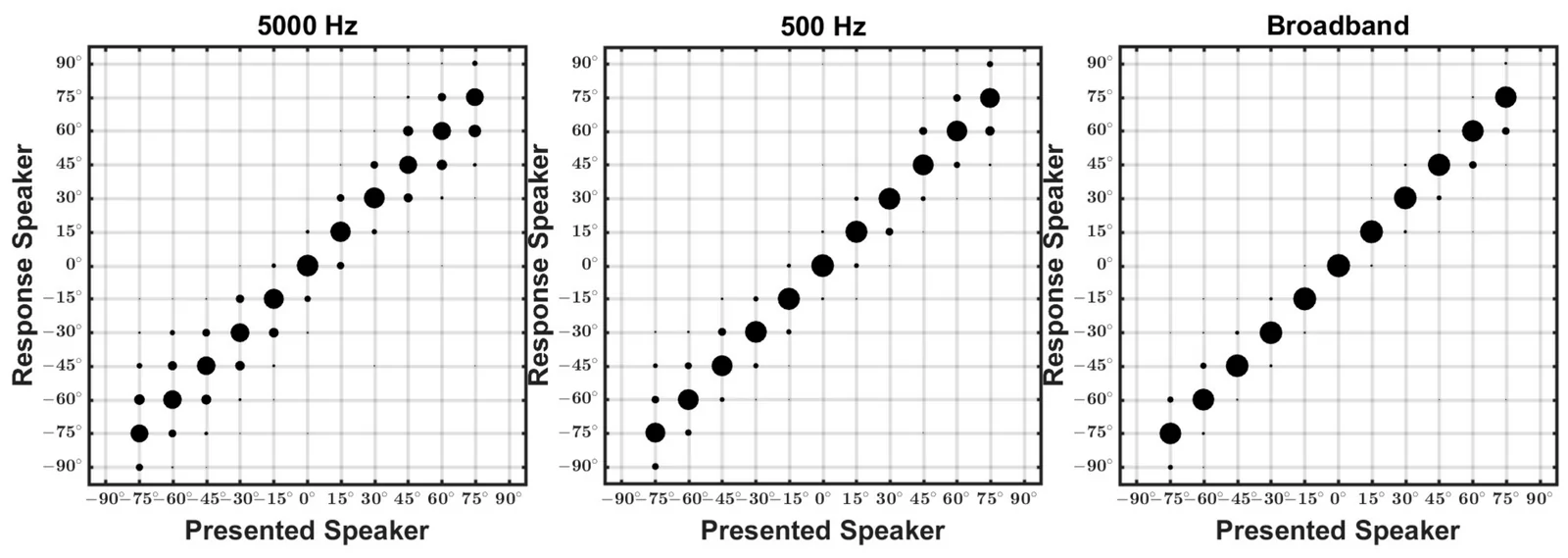
Distribution of localization responses as a function of presented loudspeaker location for the three types of noise stimuli. Bubble size represents the proportion of responses for each presented–response location pair.

Figure 4 shows the distribution of localization responses as a function of presented speaker location for the three stimulus conditions (5000 Hz, 500 Hz, and broadband). Overall, responses in all three conditions followed a clear monotonic relationship with presented azimuth, with the largest bubbles concentrated along the main diagonal. Localization performance was most accurate in the broadband condition, as evidenced by the tight clustering of large response bubbles along the diagonal across the full range of azimuths (−75° to +75°). Responses closely matched the presented speaker locations with minimal spread to adjacent positions, indicating high precision and low variability. In the 500 Hz condition, responses also followed the diagonal trend, indicating that listeners were able to reliably use low-frequency binaural cues (primarily ITDs) for localization. However, compared to the broadband condition, there was slightly greater dispersion around the diagonal, particularly at more lateral positions (±60° to ±75°). Localization in the 5000 Hz condition showed the greatest spread of responses relative to the other conditions. Although the dominant responses still aligned with the diagonal, indicating overall correct localization trends, the distribution of bubbles was more diffuse, with larger off-diagonal responses compared to both the 500 Hz and broadband conditions. Overall, the pattern of results indicates that listeners were generally able to correctly map perceived sound location to the corresponding loudspeaker position across the three noise conditions.

**Figure 5:**
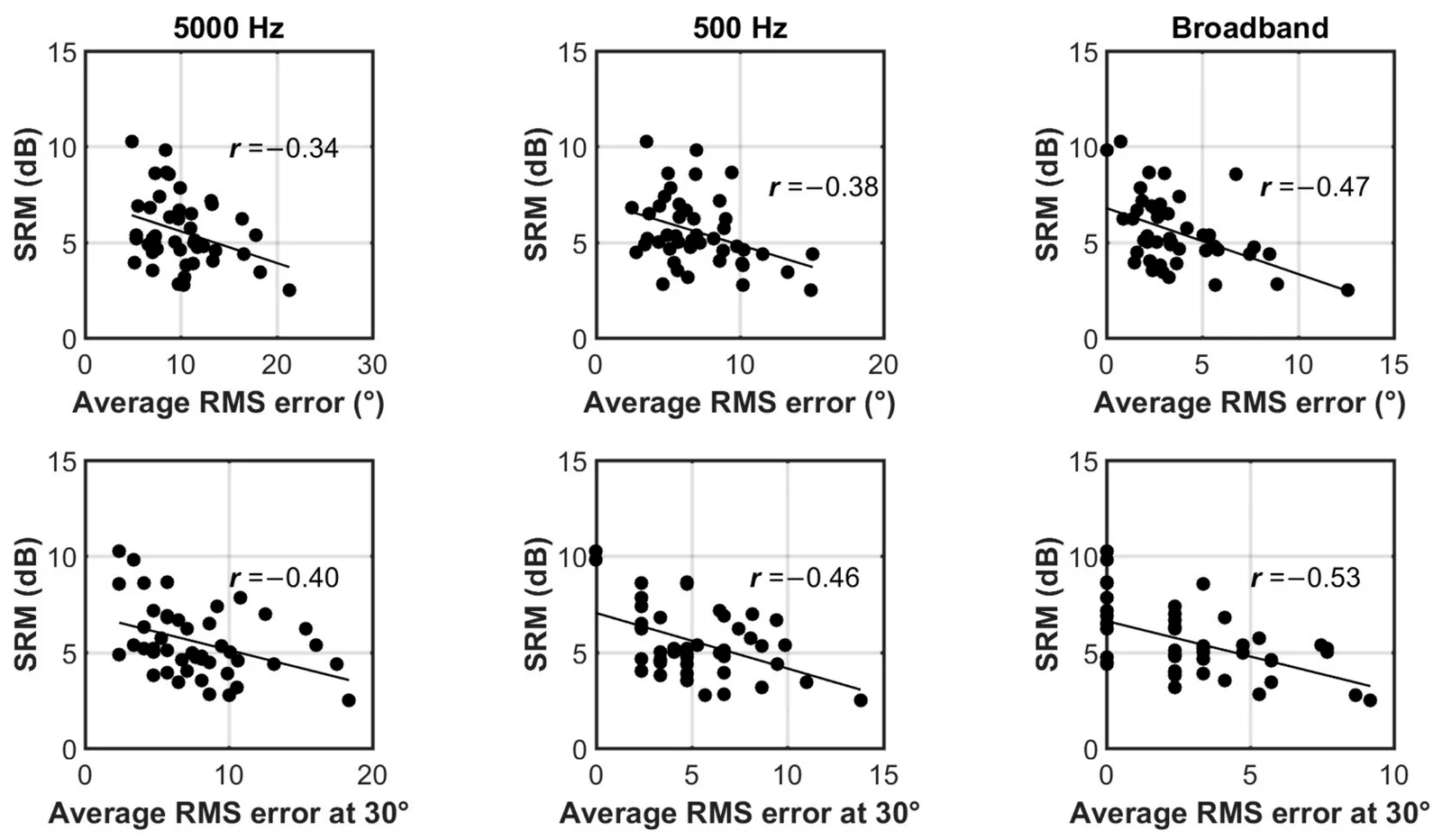
The top row shows the scatter plot between SRM and average RMS error across all presented loudspeakers for the three types of noise stimuli, while the bottom row shows the scatter plot between SRM and average RMS error at 30° azimuth. All correlations were significant at the *p* < 0.05 level. The line within each panel indicates the best fit line to the data.

Figure 5 illustrates the relationship between SRM and localization acuity, quantified as RMS error, for the three noise conditions (high-frequency, low-frequency, and broadband). Across all conditions, a negative relationship was observed between RMS error and SRM, indicating that listeners with better localization acuity (i.e., lower RMS error) tended to exhibit greater spatial release from masking. When localization acuity was calculated across all speaker locations, the strength of this relationship was moderate for all stimulus types. Specifically, SRM was negatively correlated with RMS error for the high-frequency condition (*r* = −0.34, 95% CI [−0.58, −0.05]), the low-frequency condition (*r* = −0.38, 95% CI [−0.61, −0.10]), and the broadband condition (*r* = −0.47, 95% CI [−0.67, −0.21]). A numerically larger relationship emerged when localization acuity was restricted to performance at ±30° azimuth. In this case, the magnitude of the correlations increased for all stimulus conditions, with *r* = −0.40 (95% CI [−0.62, −0.12]) for the high-frequency condition, *r* = −0.46 (95% CI [−0.66, −0.19]) for the low-frequency condition, and *r* = −0.53 (95% CI [−0.71, −0.28]) for the broadband condition. Dependent correlations sharing one variable (SRM) were compared using Steiger’s z-test for correlated correlations. The resulting analyses indicated that the differences in correlations between average RMS errors and average RMS errors at ±30° azimuth with SRM were not statistically significant. Thus, the present data do not provide evidence that localization performance at ±30° is a stronger predictor of SRM than overall localization performance.

## 4. Discussion

The present study examined the relationship between localization acuity and spatial release from masking (SRM) by systematically manipulating the spectral content of localization stimuli and relating performance to speech-in-noise outcomes in a multitalker environment. Several important findings emerged. The results revealed three primary findings: (1) localization acuity varied as a function of stimulus bandwidth, with broadband noise yielding the highest accuracy, followed by low-frequency and high-frequency noise; (2) listeners demonstrated robust SRM when spatial cues were available; and (3) localization acuity was moderately associated with SRM across stimulus conditions, with numerically larger—but not statistically different—correlations observed for broadband stimuli and localization measures obtained at ±30° azimuth. Together, these findings provide new insight into the extent to which basic spatial resolution abilities contribute to functional listening in complex acoustic environments.

### 4.1. Integration of Binaural Cues and Localization Acuity

The superior localization performance observed for broadband stimuli reinforces the well-established view that accurate spatial perception depends on the integration of multiple cue types across frequency. Broadband signals provide simultaneous access to interaural time differences (ITDs), interaural level differences (ILDs), and spectral shape cues, allowing the auditory system to construct robust and redundant spatial representations [1,2]. In contrast, narrowband signals limit the availability of these cues, forcing listeners to rely on a restricted subset of spatial information.

The finding that low-frequency localization (500 Hz) outperformed high-frequency localization (5000 Hz) is consistent with classical binaural hearing theory. ITDs, which dominate at low frequencies, provide precise and unambiguous cues for azimuthal localization, particularly in the frontal field [10]. In contrast, ILDs, which dominate at high frequencies, are more susceptible to variability due to head-shadow effects and may be less reliable in isolation [25]. Furthermore, high-frequency localization relies more heavily on spectral cues, which can be ambiguous for narrowband stimuli lacking sufficient bandwidth [29].

These findings are also in agreement with free-field localization studies demonstrating that localization accuracy improves with increasing spectral bandwidth and cue availability [16]. The bubble plot patterns observed here—specifically the broader spread of responses in the high-frequency condition—mirror the increased spatial uncertainty reported in studies where binaural or spectral information is degraded [6]. The present results are consistent with prior work demonstrating that spatial acuity critically depends on both the fidelity and integration of binaural cues in realistic listening conditions [1,2,6,16].

### 4.2. Spatial Release from Masking

Listeners in the present study exhibited robust SRM (≈5.5 dB), consistent with previous reports for young normal-hearing listeners [6,7,17]. The lower thresholds observed in the spatially separated condition indicate improved speech recognition performance and reflect the benefit provided by spatial separation of the competing maskers. Spatial separation between the target and maskers improves speech perception through both energetic and informational mechanisms. Energetic unmasking arises from head-shadow effects and binaural disparities in signal-to-noise ratio, whereas informational masking is reduced when spatial cues facilitate perceptual segregation of competing streams [9,19]. Importantly, SRM reflects not only peripheral encoding but also central auditory processes related to auditory scene analysis and selective attention [11]. The ability to form stable spatial representations and use them to guide attention is critical for extracting target speech in complex environments.

It is important to note that the current study employed a free-field loudspeaker array in a sound-treated booth, which provides a highly ecologically valid representation of real-world listening environments. Free-field presentation preserves natural binaural cues, including head-related transfer functions, interaural differences, and spectral shaping effects, thereby allowing listeners to utilize spatial information in a manner that closely approximates everyday listening. While previous studies [17] have shown strong agreement in SRM estimates across headphone-based and free-field testing, free-field configurations remain the gold standard for assessing spatial hearing because they capture the full fidelity of acoustic spatial cues without reliance on virtual rendering. Consequently, the SRM values obtained in the present study can be interpreted as reflecting listeners’ true spatial listening capabilities under realistic acoustic conditions. This methodological consideration strengthens the ecological validity of the observed relationship between localization acuity and SRM, as both measures were obtained using naturalistic free-field cues that closely mirror the demands of real-world auditory scene analysis.

### 4.3. Relationship Between Localization Acuity and SRM

A central goal of this study was to determine whether localization acuity predicts SRM. The results revealed consistent negative correlations between RMS error and SRM across all stimulus conditions, indicating that listeners with better spatial resolution tend to benefit more from spatial separation. However, the magnitude of these correlations was moderate, suggesting that localization acuity accounts for only part of the variability in SRM.

This finding aligns with a growing body of literature reporting weak-to-moderate associations between spatial hearing measures and speech-in-noise performance [12,14]. For example, ref. [12] showed that binaural sensitivity and cognitive abilities both contribute to speech perception in spatialized environments, while [14] reported dissociations between localization performance and speech-in-noise outcomes in certain populations. Moreover, different measures of spatial hearing (e.g., localization, lateralization, and speech perception) do not necessarily correlate strongly within individuals, highlighting that these tasks tap into overlapping but distinct auditory processes [4,30,31].

The broadband condition yielded numerically larger correlations with SRM. However, because differences among the correlation coefficients were not statistically significant, these observations should be interpreted cautiously. When listeners have access to the full complement of spatial cues, localization performance becomes a more reliable indicator of the quality of internal spatial representations, which in turn supports more effective speech segregation. This interpretation is consistent with neurophysiological models suggesting that spatial hearing arises from distributed processing across frequency channels and hierarchical integration within the auditory pathway [10,32].

The correlations between localization acuity and SRM were numerically greater when localization performance was evaluated at ±30° azimuth, a spatial location directly relevant to the SRM task. However, statistical comparisons of the correlation coefficients were not significant. Consequently, while the observed pattern is consistent with the possibility that task-relevant spatial locations may play an important role in SRM, additional studies with larger samples are needed to determine whether these numerical differences reflect a reliable effect. Although localization performance at ±30° azimuth yielded numerically larger correlations with SRM, the absence of statistically significant differences means that the present results cannot establish a preferential role of task-relevant spatial locations. This result highlights the importance of task-relevant spatial regions when linking spatial hearing measures to functional outcomes and aligns with ecological models of auditory perception, which emphasize that spatial hearing is context-dependent and optimized for behaviorally relevant regions of space [5]. It also supports the idea that global summary measures of localization (e.g., overall RMS error) may obscure meaningful relationships that are more apparent when analyzing performance in specific spatial regions.

An additional finding was that localization acuity was associated with speech-identification performance in the spatially separated condition but not in the colocated condition. The correlations between localization measures and speech identification thresholds are shown in Table 1. This pattern is consistent with the notion that localization and SRM share underlying mechanisms related to the encoding and utilization of binaural spatial cues. In the spatially separated condition, listeners must exploit interaural time differences, interaural level differences, and other spatial attributes to perceptually segregate the target speech from competing maskers, making the fidelity of spatial cue processing directly relevant to performance [6,7,11]. In contrast, when target and masker are colocated, spatial cues provide little benefit for source segregation because both signals originate from the same location. Under these circumstances, performance is likely influenced more strongly by factors such as audibility, speech processing ability, susceptibility to informational masking, and cognitive resources than by localization acuity itself [8,9,15,20]. The absence of a significant relationship between localization performance and colocated thresholds therefore suggests that localization acuity contributes specifically to the ability to utilize spatial separation rather than to speech recognition performance in general. This interpretation is consistent with previous work indicating that different spatial-hearing measures share some common underlying binaural processes while nevertheless reflecting partially distinct perceptual and cognitive mechanisms [12,14,31].

Another consideration is the potential contribution of binaural unmasking mechanisms that are commonly assessed using the binaural masking level difference (MLD) paradigm. Although MLD and free-field localization both depend on binaural processing, they assess somewhat different aspects of spatial hearing. Localization requires the accurate encoding and interpretation of spatial cues to determine sound-source direction, whereas MLD primarily reflects the auditory system’s ability to use interaural differences to improve signal detection in noise through binaural interaction mechanisms [19]. Previous studies have reported that MLD measures can be relatively independent of localization performance, suggesting that different components of binaural processing contribute to distinct spatial-hearing tasks [10,11]. Because SRM reflects both binaural unmasking and higher-order spatial segregation processes [8,9], it is possible that MLD performance may explain additional variance in SRM beyond that accounted for by localization acuity alone. The moderate correlations observed in the present study are consistent with the idea that localization represents only one component of a broader spatial-hearing system. Future studies incorporating both localization measures and MLD paradigms may help distinguish the relative contributions of spatial cue encoding, binaural unmasking, and higher-level auditory scene analysis processes to individual differences in SRM [19,20,23].

### 4.4. Contributions of Cognitive and Attentional Processes

One possible explanation for the moderate correlations observed in the present study is that higher-level cognitive factors may contribute to SRM performance. However, these variables were not measured directly and therefore this interpretation remains speculative. While accurate spatial encoding is necessary for segregating sound sources, it is not sufficient. Listeners must also be able to selectively attend to the target stream and suppress competing information. A considerable body of literature has demonstrated that cognitive factors such as attention and working memory contribute significantly to speech perception in noise. SRM is influenced by individual differences in spatial attention and susceptibility to informational masking [15,33,34,35,36]. One possible interpretation is that listeners with similar localization acuity could differ in their ability to utilize spatial information in complex listening environments. However, because cognitive abilities were not measured in the present study, this explanation remains speculative. Listeners with similar localization acuity may differ in their ability to effectively utilize spatial cues for source segregation. Thus, the present findings support a framework in which localization acuity represents a foundational sensory ability that interacts with higher-level perceptual and cognitive processes to determine SRM performance [23,37].

### 4.5. Clinical and Theoretical Implications

From a theoretical perspective, the results reinforce the view that spatial hearing is not a unitary construct but rather a combination of interrelated processes. Localization acuity, while informative, captures only one aspect of spatial hearing and cannot fully predict functional outcomes such as SRM. From a clinical standpoint, these findings suggest that assessments of spatial hearing should incorporate both localization tasks and speech-in-noise measures to obtain a comprehensive understanding of functional listening ability. The stronger relationship observed for broadband and lateral localization conditions indicates that ecologically valid stimuli and task-relevant spatial configurations may improve the predictive value of clinical tests.

## 5. Limitations

The present study focused on young normal-hearing listeners, limiting the generalizability of the findings. The relatively homogeneous sample of young normal-hearing female listeners may have restricted performance variability, potentially reducing the magnitude of observable correlations, as anatomical differences can influence the available range of binaural cues. Future work should examine these relationships in older adults and in individuals with hearing loss, where deficits in binaural processing and cognitive function may alter the relationship between localization and SRM [3]. Although participants were instructed to maintain a fixed head orientation, no chin rest or head-tracking system was used. Small head movements may have introduced dynamic spatial cues that could have influenced localization performance. Future studies may benefit from mixed-effects modeling approaches that incorporate trial-level responses while accounting for participant-specific variability and repeated observations. Additionally, future studies could incorporate dynamic listening conditions, including head movements and moving sound sources, which are known to enhance spatial perception and may provide additional insight into real-world listening behavior [38,39]. Also, SRM was measured using only a single masker configuration (±30°). Future research should include multiple spatial separations to determine whether the relationship between localization acuity and SRM varies as a function of spatial geometry.

## 6. Conclusions

In summary, the present study demonstrates that localization acuity is systematically related to spatial release from masking, with better spatial resolution associated with greater benefit from spatial separation. However, this relationship is moderate and depends on both stimulus bandwidth and spatial context. These findings indicate that localization acuity contributes to individual differences in SRM but also suggest that additional auditory and non-auditory factors influence spatial hearing performance.

## Figures and Tables

**Table 1:** Correlations and their corresponding 95% confidence intervals between localization acuity (RMS error) and speech identification thresholds in the colocated and spatially separated conditions, as well as spatial release from masking (SRM).

	Colocated			Separated			SRM		
	*r*	95% CI		*r*	95% CI		*r*	95% CI	
		*LL*	*UL*		*LL*	*UL*		*LL*	*UL*
Broadband RMS ±30°	−0.03	−0.32	0.26	**0.49**	0.23	0.69	**−0.53**	−0.71	−0.28
500 Hz RMS ±30°	0.04	−0.26	0.33	**0.45**	0.18	0.66	**−0.46**	−0.66	−0.19
5000 Hz RMS ±30°	0.01	−0.28	0.31	**0.38**	0.09	0.61	**−0.40**	−0.62	−0.12

*Note: n* = 45. CI = confidence interval; *LL* = lower limit; *UL* = upper limit. Bolded correlations are significant at *p* < 0.05 level.

## Data Availability

The data presented in this study are available upon request from the corresponding author due to their inclusion in a larger dataset that is currently being analyzed.
